# The sarcopenia–pain connection: why mechanisms matter for patient care?

**DOI:** 10.3389/fpain.2026.1759943

**Published:** 2026-03-30

**Authors:** Giovanni Iolascon, Antimo Moretti

**Affiliations:** Multidisciplinary Department of Medical and Surgical Specialties and Dentistry, University of Campania “Luigi Vanvitelli”, Naples, Italy

**Keywords:** aging, exercise, nutrition, pain, rehabilitation, sarcopenia

## Abstract

Sarcopenia and pain are two highly prevalent conditions in aging populations, each exerting profound effects on mobility, independence, and quality of life. Emerging evidence demonstrates that these conditions are not merely coincidental but are closely interconnected through shared biological, mechanical, and neurophysiological pathways. Pain reduces physical activity, accelerates muscle wasting, and fosters functional decline, while sarcopenia increases vulnerability to painful syndromes such as osteoarthritis, fragility fractures, and low back pain. This bidirectional relationship is further amplified by chronic low-grade inflammation (“inflammaging”), mitochondrial dysfunction, and central sensitization, creating a vicious cycle of musculoskeletal fragility and disability. Despite robust epidemiological data, current diagnostic frameworks for sarcopenia fail to integrate pain dimensions, risking misclassification and underestimation of disease burden. Novel screening approaches that combine anthropometric measures with validated pain assessments may improve case finding and clinical management. This narrative review synthesizes epidemiological insights, mechanistic links, and diagnostic challenges, and advocates for integrated strategies that simultaneously target muscle health and pain management. Recognizing pain as both a determinant and consequence of sarcopenia is essential to advancing prevention, rehabilitation, and multidisciplinary care in older adults.

## 1 Background

Sarcopenia, defined as the age-related loss of skeletal muscle mass and function, is now recognized as a major geriatric syndrome with significant implications for mobility, independence, and survival (1). According to European Working Group on Sarcopenia in Older People (EWGSOP), sarcopenia is classified into two categories: primary when the only apparent cause is age itself, and secondary when one or more identifiable factors contribute to muscle loss, such as physical inactivity, nutritional deficiencies, or chronic illnesses (2). Often, in older individuals, the causes are multifaceted, making it challenging to assign a strictly exclusive diagnosis of either primary or secondary sarcopenia.

The European Working Group on Sarcopenia in Older People (EWGSOP2) revised consensus emphasized that sarcopenia should not be understood merely as a decline in muscle mass, but also as impaired strength and performance, which are the primary drivers of adverse outcomes in older adults (1). More recently, the Global Leadership Initiative in Sarcopenia (GLIS), proposed the first global conceptual definition of sarcopenia, encompassing low muscle mass, reduced muscle strength, and impaired muscle-specific strength, while identifying compromised physical performance as a key clinical outcome (3).

Physical performance encompasses a range of functions, such as mobility, balance, and gait speed, all of which may be substantially influenced by the presence of pain (4). Musculoskeletal (MSK) pain is one of the most frequent and disabling symptoms in older adults and is a leading contributor to years lived with disability (YLDs) worldwide (5). Pain may arise from multiple sources, including skeletal muscle, which is richly innervated by nociceptive afferents. Thinly myelinated group III (Aδ) and unmyelinated group IV (C) fibers respond to high-threshold mechanical, chemical, and metabolic stimuli, transmitting signals to the spinal cord and making muscle a potential generator of deep somatic pain (6).

Chronic pain and sarcopenia are closely interrelated in older adults, often coexisting and reinforcing each other through shared biological and functional pathways (7). Pain can reduce physical activity, leading to disuse, muscle wasting, and functional decline, while sarcopenia may increase susceptibility to musculoskeletal conditions such as osteoarthritis, vertebral fragility fractures, and low back pain (8). This bidirectional relationship creates a vicious cycle that amplifies musculoskeletal fragility, disability, and reduced quality of life (9). Evidence suggests that pain not only correlates with prevalent sarcopenia but may also predict its onset, while sarcopenia increases vulnerability to chronic musculoskeletal pain (10, 11). This bidirectional relationship between pain and sarcopenia underscores the need for integrated strategies that simultaneously target pain management and muscle health in aging populations.

This review synthesizes current evidence on the interplay between sarcopenia and pain, with a particular emphasis on epidemiological insights, underlying mechanisms, diagnostic challenges and opportunities, and therapeutic strategies.

## 2 Epidemiological insights

Epidemiological evidence consistently supports a significant association between sarcopenia and chronic musculoskeletal pain across diverse populations and healthcare systems. Large-scale observational studies, including both cross-sectional and longitudinal cohort designs, have demonstrated that older adults with pain exhibit a higher prevalence and incidence of sarcopenia (12).

Cross-sectional analysis, such as those derived from The China Health and Retirement Longitudinal Study (CHARLS) demonstrated that sarcopenia and pain both reduce the probability of successful aging, defined according to the Rowe and Kahn's multidimensional model, which considers the absence of major diseases, disability, cognitive impairment, depression and poor social engagement (13). Authors analyzed data from 4,280 Chinese adults aged over 60 years, with 1,822 followed up after two years, reporting that sarcopenia was associated with a 47.7% lower likelihood of successful aging compared to those without sarcopenia (OR: 0.523, 95% CI: 0.315–0.869), pain reduced the likelihood of successful aging by 61.2% (OR: 0.388, 95% CI: 0.251–0.600), while coexisting sarcopenia and pain further decreased the likelihood by 73.2% (OR: 0.268, 95% CI: 0.108–0.759).

Longitudinal cohort studies offer stronger inferential value. The English Longitudinal Study of Ageing (ELSA) followed a large representative cohort of older adults over ten years, showing that baseline pain, particularly when moderate to severe and affecting multiple sites such as the low back and lower limbs, significantly increased the risk of developing sarcopenia (OR = 1.46: 95% CI: 1.18–1.82). This association persisted after adjusting for confounders including age, sex, comorbidities, and lifestyle factors, suggesting that pain itself contributes to muscle wasting. This study provides robust cohort-level evidence that pain should be considered a modifiable risk factor in sarcopenia prevention frameworks, with implications for screening, early intervention, and multidisciplinary rehabilitation approaches (10). Similarly, data from low- and middle-income countries showed that the association between pain and sarcopenia is consistent across different health systems and cultural contexts, confirming its global relevance. Smith et al. (14) analyzed nationally representative data from the WHO Study on global AGEing and adult health (SAGE), including older adults across six LMICs, reporting that older adults experiencing pain had a markedly higher prevalence of sarcopenia, even after adjusting for sociodemographic and health-related confounders. Moreover, authors identified sedentary behavior, depressive symptoms, and disability may partially explain the observed association, pointing to complex biopsychosocial pathways.

Otherwise, several disease-specific epidemiological studies suggest that sarcopenia may contribute to pain vulnerability, particularly in osteoarthritis and low back pain. In postmenopausal women, sarcopenia and sarcopenic obesity were associated with greater joint pain and more severe radiographic and clinical features of osteoarthritis (15). Notably, sarcopenic obesity exerted the strongest effect, suggesting that the coexistence of muscle weakness and fat accumulation amplifies mechanical stress and inflammatory pathways within joints. Moreover, this study suggests that sarcopenia contributes to pain not merely through reduced mobility but also via pathophysiological mechanisms involving inflammation, altered biomechanics, and metabolic dysregulation.

In the GAINA study, participants with sarcopenia had a significantly higher prevalence of chronic low back pain compared to those without sarcopenia, even after adjusting for age, sex, and comorbidities, suggesting that reduced muscle mass and strength may compromise spinal support and stability, thereby increasing susceptibility to pain. These findings highlight sarcopenia as a potential risk factor for spinal pain conditions, reinforcing the concept that muscle wasting is not only a marker of frailty but also a contributor to specific pain syndromes (16).

Another Japanese study identified correlations between muscle wasting, chronic musculoskeletal pain, and central sensitization in community-dwelling older adults. Imai et al. (17) reported that older adults with chronic pain were more likely to present with sarcopenia, and this association was significantly mediated by central sensitization. In particular, the pressure pain threshold (PPT) was significantly associated with the presence of chronic pain with sarcopenia or presarcopenia, suggesting that abnormal pain processing may amplify the impact of muscle loss on functional outcomes, highlighting a pain–sarcopenia link that is not merely mechanical but also neurophysiological.

Although epidemiological studies consistently demonstrate an association between sarcopenia and pain across diverse populations, most available data derive from cross-sectional or observational cohort designs. Therefore, causality cannot be definitively established, and residual confounding factors, including comorbidities and lifestyle variables, may influence the observed relationships.

## 3 Pathophysiological links

The biological plausibility of the sarcopenia–pain connection is supported primarily by experimental and translational evidence. Mechanistic insights derive from molecular studies, animal models, and neurophysiological investigations, which collectively elucidate how aging-related alterations may simultaneously affect muscle integrity and nociceptive processing. However, while these mechanisms provide biological coherence, their hierarchical contribution and causal relevance in older people remain not fully established.

Skeletal muscle tissue is richly innervated by a variety of sensory afferents capable of detecting mechanical, chemical, and metabolic changes (18). Group III (A*δ*) and group IV (C) muscle afferents are predominantly responsible for nociceptive signaling from muscles (19). Group III fibers are thinly myelinated and respond primarily to high-threshold mechanical stimuli, while group IV fibers are unmyelinated and sensitive to mechanical, thermal, and chemical changes within the muscle, including metabolites produced during contraction or ischemia. These afferents transmit signals via the dorsal root ganglia to the spinal cord, where they synapse on second-order neurons in the dorsal horn, contributing to the perception of deep somatic pain.

Additionally, low-threshold mechanoreceptors in muscle and fascia can mediate non-painful pressure sensations, but under high-intensity or pathological stimulation, these same structures can contribute to pain perception (6). The combination of these afferent types ensures that skeletal muscle is not only a functional contractile tissue but also a potent pain generator under conditions of trauma, overuse, or inflammation.

Muscle pain differs in multiple features from pain originating in the skin or viscera. These differences involve not only the underlying pathophysiological mechanisms but also a range of subjective characteristics. As summarized by Mense (20), muscle pain is typically described as deeper, more diffuse, and harder to localize than cutaneous pain, and it more frequently manifests as referred pain. In addition, muscle pain often persists longer, is accompanied by greater autonomic responses, and is more likely to impair motor function. These features highlight that the mechanisms of cutaneous pain cannot simply be extrapolated to muscle pain, which represents a distinct neurophysiological entity with unique clinical implications.

Muscle pain can be pathogenetically classified by considering the specific pain generators within muscle tissue. It may arise from different structural components, including connective tissue elements, each capable of producing pain that can be characterized as mechanical or inflammatory. As highlighted by Mense (20), peripheral nociceptors in muscle are activated not only by mechanical strain or tissue damage but also by metabolic and chemical stimuli such as ATP release and reduced pH. These excitatory factors contribute to sustained nociceptor activity, which in turn can induce central sensitization and amplify pain perception.

In primary sarcopenia, both mechanisms of nociceptive stimulation (i.e., mechanical and/or inflammatory) might be involved. The first is related to the lowering of the mechanical stimulation threshold, as mechanoreceptors (proprioceptors) might become more sensitive and transmit pain perception (21). Indeed, the acid-sensing ion channel 3 (ASIC3), known for their pro-nociceptive role, are also expressed in non-nociceptive proprioceptors, where they contribute to proprioceptive functions (22). In addition, ASICs are found in muscle afferent neurons that mediate antinociceptive signaling (23). Consequently, the perception of acid stimuli arises from the integrated activity of diverse somatosensory neurons—including nociceptors, proprioceptors, and other neuronal subtypes—rather than from nociceptors alone. This complexity suggests that alterations in muscle microenvironment may shift the balance between protective proprioception and maladaptive nociception.

The second mechanism of nociceptive stimulation in sarcopenia might be due to increased inflammatory cytokines, a hallmark of “inflammaging” (24, 25). Chronic low-grade inflammation, or “inflammaging,” is a common denominator of both sarcopenia and pain. Experimental studies have demonstrated that elevated cytokines such as IL-6 and TNF-α contribute to muscle protein catabolism while simultaneously sensitizing nociceptors and promoting central sensitization. Elevated cytokines such as TNF-α, IL-6, IL-1β, and CRP promote protein degradation, inhibit muscle protein synthesis, and impair satellite cell–mediated regeneration, thereby accelerating muscle atrophy and weakness (26). At the cellular level, these cytokines activate NF-*κ*B signaling and upregulate muscle-specific ubiquitin ligases (MuRF-1 and Atrogin-1), thereby enhancing proteasomal degradation of myofibrillar proteins (26). In parallel, chronic inflammation disrupts satellite cell activation and increases oxidative stress, further diminishing regenerative capacity (27). Moreover, the same mediators sensitize peripheral nociceptors and amplify pain signaling. Prolonged exposure to inflammatory mediators fosters central sensitization, lowering pain thresholds and maintaining chronic pain (28).

This shared inflammatory substrate provides a compelling mechanistic bridge between muscle atrophy and persistent pain. Nevertheless, the strength of the inflammatory hypothesis remains debated. Cross-sectional data demonstrates only modest associations between systemic cytokine levels and muscle decline after adjusting for multimorbidity and lifestyle factors in older adults (29). Moreover, not all individuals with elevated inflammatory markers develop sarcopenia or chronic pain, suggesting that inflammation may function as a permissive or amplifying factor rather than a causal determinant (30).

Age-related mitochondrial dysfunction exacerbates both sarcopenia and pain (31). Impaired oxidative phosphorylation and reduced mitochondrial biogenesis lead to decreased ATP availability and increased generation of reactive oxygen species (ROS), thereby reinforcing both muscle decline and chronic pain persistence (31). While physiological concentrations of ROS are indispensable for regulating normal myocyte function, force production, and adaptive responses to exercise, their excessive accumulation—defined as oxidative stress—induces cellular damage, driving the shared pathological mechanisms in both conditions (32).

Elevated ROS levels trigger pivotal pro-inflammatory signaling cascades, most notably NF-κB activation, culminating in the enhanced expression and subsequent secretion of key inflammatory cytokines, such as TNF-α and IL-6 (33). Specifically, within the muscle interstitium, these cytokines accelerate protein catabolism via activation of the ubiquitin-proteasome system (34). This process simultaneously hinders *de novo* protein synthesis and compromises the regenerative potential of muscle stem cells (satellite cells), ultimately driving progressive muscle atrophy and functional decline (35). Concurrently, within the nervous system, these identical inflammatory mediators induce peripheral sensitization of nociceptors and facilitate central sensitization, rendering both spinal cord and brain neurons hyper-responsive to sensory stimuli, thereby sustaining persistent nociceptive states (36). Furthermore, surplus ROS directly inflict substantial oxidative damage upon crucial cellular macromolecules—proteins, lipids, and DNA—across both muscle and neural tissues. This damage critically compromises musculoskeletal integrity and contributes directly to the tissue injury and neuropathic changes underpinning chronic pain etiologies (37).

Experimental models suggest that mitochondrial-targeted antioxidants may attenuate both muscle atrophy and pain hypersensitivity (38). However, clinical translation remains limited, and human studies directly linking mitochondrial dysfunction to the coexistence of sarcopenia and chronic pain are still scarce. Thus, although biologically plausible, mitochondrial mechanisms require further longitudinal validation.

Anabolic resistance and neuromuscular degeneration are commonly described pathogenic contributors to sarcopenia. Aging muscle exhibits reduced responsiveness to anabolic stimuli, largely mediated by impaired IGF-1/PI3K/Akt/mTOR signaling. Reduced activation of mTOR diminishes protein synthesis and accelerates muscle loss (39). Chronic pain, through physical inactivity, stress-mediated endocrine changes, and altered motor unit recruitment, may further exacerbate anabolic resistance (40).

In parallel, age-related degeneration of the neuromuscular junction (NMJ) contributes to motor unit remodeling and partial denervation. Experimental evidence indicates that impaired reinnervation capacity precedes overt muscle fiber atrophy (41). Emerging neurophysiological data suggests that altered motor control and cortical reorganization in chronic pain states may further disrupt neuromuscular efficiency (42).

**Figure 1:**
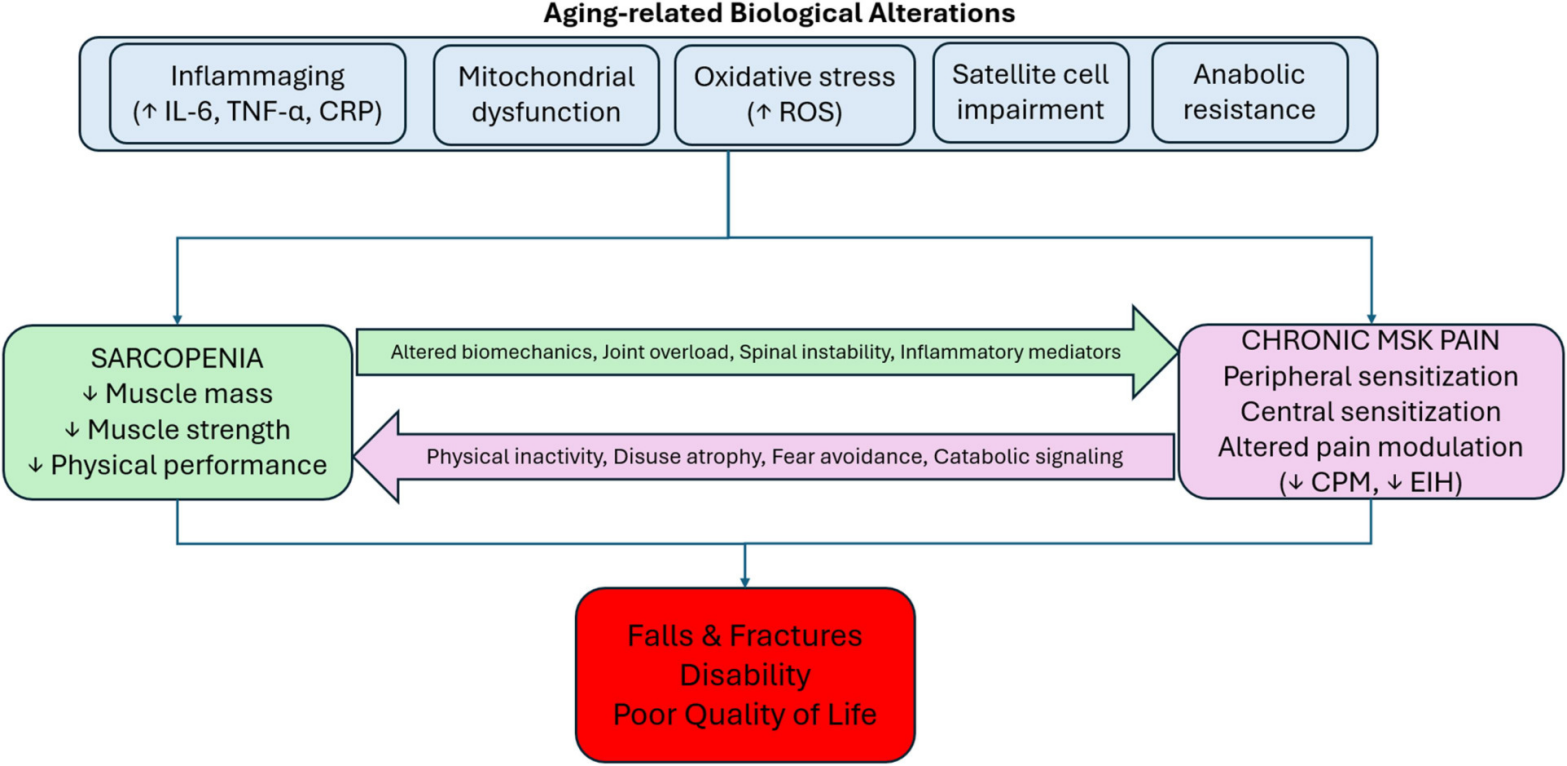
Conceptual framework summarizing the biological, mechanical, and neurophysiological mechanisms underpinning the bidirectional sarcopenia–pain relationship.

However, direct molecular evidence linking mTOR dysregulation or NMJ degeneration to increased pain susceptibility in older people remains limited. Much of the support derives from animal or indirect translational models, and whether these represent causal drivers or parallel aging processes remains uncertain.Together, the interplay between sarcopenia and pain reflects a convergence of mechanical, inflammatory, mitochondrial, neurobiological, and psychosocial processes (9). These mechanisms create a vicious cycle in which inflammation, oxidative stress, anabolic resistance and neurophysiological sensitization mutually intensify muscle wasting and chronic pain, underscoring the need for interventions that target shared pathways to disrupt this continuum in older adults (Figure 1).

Much of the mechanistic understanding derives from experimental and translational studies, including animal models and molecular investigations. While these findings provide biological plausibility for the sarcopenia–pain connection, their direct clinical applicability in older adults remains to be fully elucidated.

## 4 Diagnostic issues and potential solutions

The diagnosis of sarcopenia remains challenging, particularly when considering its complex relationship with pain. Current international definitions, including the European Working Group on Sarcopenia in Older People 2 (EWGSOP2) and the Asian Working Group for Sarcopenia (AWGS), prioritize low muscle strength as the primary diagnostic criterion, confirmed by reduced muscle mass, with impaired physical performance used to determine severity (1, 3, 43). Screening tools such as SARC-F are recommended for case finding, followed by objective assessment of handgrip strength, chair stand performance, and body composition analysis (DXA, BIA, CT, or MRI). Notably, none of these frameworks formally incorporate pain assessment within their diagnostic algorithms (1, 3, 43). This omission is problematic because chronic musculoskeletal pain both masks and mimics functional decline, as patients may score poorly on muscle function assessment due to pain-related limitations rather than true muscle weakness, leading to potential misclassification (44).

Conversely, individuals with sarcopenia may present with persistent pain that is overlooked in diagnostic frameworks, despite evidence that pain characteristics predict incident sarcopenia and that sarcopenia itself increases the risk of chronic pain syndromes (10, 15, 16, 45).

The lack of integration between pain assessment and sarcopenia screening tools such as SARC-F or SARC-CalF highlights a diagnostic gap, where functional impairment is interpreted without accounting for the confounding role of pain (46). Addressing this gap requires a more holistic approach that incorporates pain dimensions into sarcopenia clinical issues, ensuring that diagnostic criteria reflect the bidirectional interplay between muscle decline and musculoskeletal pain. In this context, a prospective study included 172 patients with chronic musculoskeletal pain, all of whom completed the SARC-F questionnaire, the SARC-CalF (which integrates calf circumference), and validated pain measures such as the numeric rating scale (NRS) and the pain disability assessment scale (PDAS), reporting that SARC-F alone suffers from low sensitivity, meaning it risks missing a substantial proportion of sarcopenia cases (47). By contrast, SARC-CalF improved sensitivity while maintaining acceptable specificity, offering a more balanced screening approach. The addition of calf circumference provided an objective anthropometric measure that mitigated the confounding effect of pain on functional performance. This refinement is particularly relevant in chronic pain populations, where subjective functional decline may not accurately reflect muscle status. This study highlighted the bidirectional relationship between pain and sarcopenia. However, the role of SARC-CalF in people with musculoskeletal pain is supported by a cross-sectional study with small sample size, limiting causal inference and predictive validity. Furthermore, cut-off values for calf circumference vary across populations and ethnic groups, which may affect generalizability. Prospective studies evaluating SARC-CalF as a predictor of sarcopenia, disability, or mortality remain limited, underscoring the need for longitudinal investigations.

Other novel indices have shown promise in identifying sarcopenia in older adults with musculoskeletal pain, particularly chronic low back pain (48). The evaluation of the waist–calf circumference ratio (WCR) in older patients with chronic low back pain offers an interesting perspective, considering that calf circumference alone provides a proxy for peripheral muscle mass, yet it fails to capture the broader imbalance between muscle loss and central adiposity that often accompanies aging and chronic pain. In a recent study, Kim et al. (48) revealed that higher WCR values were significantly associated with sarcopenia, and these patients also reported greater pain intensity and disability. Therefore, WCR emerges as a simple, inexpensive, and clinically feasible tool that can be applied in routine practice, bridging the gap between complex diagnostic frameworks and bedside screening. However, the study of Kim et al. is a retrospective analysis on a single-country cohort, potentially limiting external validity. Prospective investigations assessing predictive value of WCR for sarcopenia in people with musculoskeletal pain are lacking and standardized cut-off thresholds have not yet been established, which may constrain clinical implementation at this stage.

Traditional imaging (DXA, CT, MRI) allows quantification of muscle mass but fails to capture pain-related disability in patients with sarcopenia. Importantly, muscle strength, rather than mass, is more closely associated with pain intensity and quality of life. Tanaka et al. (49) explored the clinical impact of sarcopenia in 1,039 elderly patients with spinal pain, revealing that muscle weakness were associated with significantly worse spinal sagittal alignment, reduced trunk muscle mass, higher low back pain scores, and poorer health-related quality of life compared with normal and pre-sarcopenia patients. In contrast, patients with pre-sarcopenia maintained relatively preserved spinal alignment and clinical outcomes, underscoring that muscle weakness is the critical determinant of disability rather than muscle mass alone.

The use of functional tests for diagnosing sarcopenia is often affected by pain. Evidence indicates that handgrip strength may identify sarcopenia more effectively than the chair stand test in patients with musculoskeletal pain. Older adults are frequently unable to perform the chair stand test due to pain, balance deficits, or comorbidities, and those who attempt it may produce results confounded by factors unrelated to muscle strength, such as joint stiffness or fear of falling. By contrast, handgrip strength is a more feasible and reliable measure in this clinical setting: it can be administered quickly, requires minimal mobility, and provides a direct assessment of muscle strength that is less influenced by pain or functional impairments. The RESORT study demonstrates that reliance on the chair stand test risks underdiagnosing sarcopenia in patients unable to complete the task, thereby excluding some of the most vulnerable individuals from appropriate recognition and intervention. Handgrip strength, on the other hand, offers broader applicability and greater diagnostic accuracy, aligning more closely with the realities of geriatric rehabilitation practice (50).

The absence of pain assessment in sarcopenia diagnostic criteria represents a missed opportunity. Evidence suggests that pain should be considered both a clinical marker of sarcopenia and a modifiable target for early intervention. In chronic pain populations, documentation of pain intensity and interference at the time of functional testing may improve interpretability and reduce misclassification of sarcopenia severity (Table 1).

**Table 1:** Comparison of screening and functional assessment tools for sarcopenia in the context of chronic pain.

Tool	Type	Advantages	Limitations	Feasibility in chronic pain populations
SARC-F	Questionnaire (5 items)	Simple, rapid, no equipment required; high specificity	Low sensitivity; may miss early sarcopenia; influenced by subjective perception	High feasibility; however, responses may be confounded by pain-related disability rather than true muscle weakness
SARC-CalF	Questionnaire + calf circumference	Improved sensitivity compared to SARC-F; inexpensive; incorporates muscle mass proxy	Cut-off variability across populations; supported by cross-sectional investigation.	Moderate–high feasibility; calf circumference unaffected by acute pain, but functional items may still reflect pain interference
Waist–calf ratio (WCR)	Anthropometric index	Simple and low-cost	No longitudinal validation; lack of standardized cut-offs.	High feasibility; useful in pain populations with obesity; does not assess strength or performance
Handgrip strength	Objective strength test	Core criterion in EWGSOP2; reproducible; prognostic value	May be influenced by upper limb pain or osteoarthritis; requires dynamometer	Generally feasible; interpret cautiously in patients with hand/shoulder pain
Chair stand test	Functional lower limb strength	Reflects real-world lower limb function; widely used	Performance may be limited by knee/hip/back pain rather than muscle weakness	Reduced feasibility in severe musculoskeletal pain; pain documentation recommended during testing
Gait speed	Physical performance	Strong predictor of disability and mortality; standardized measure in sarcopenia	Influenced by pain, balance, fear of movement	Feasible but pain may confound interpretation; pain phenotyping enhances contextual accuracy

Future guidelines might incorporate pain assessment alongside muscle strength and performance for a more comprehensive diagnostic framework.

**Figure 2:**
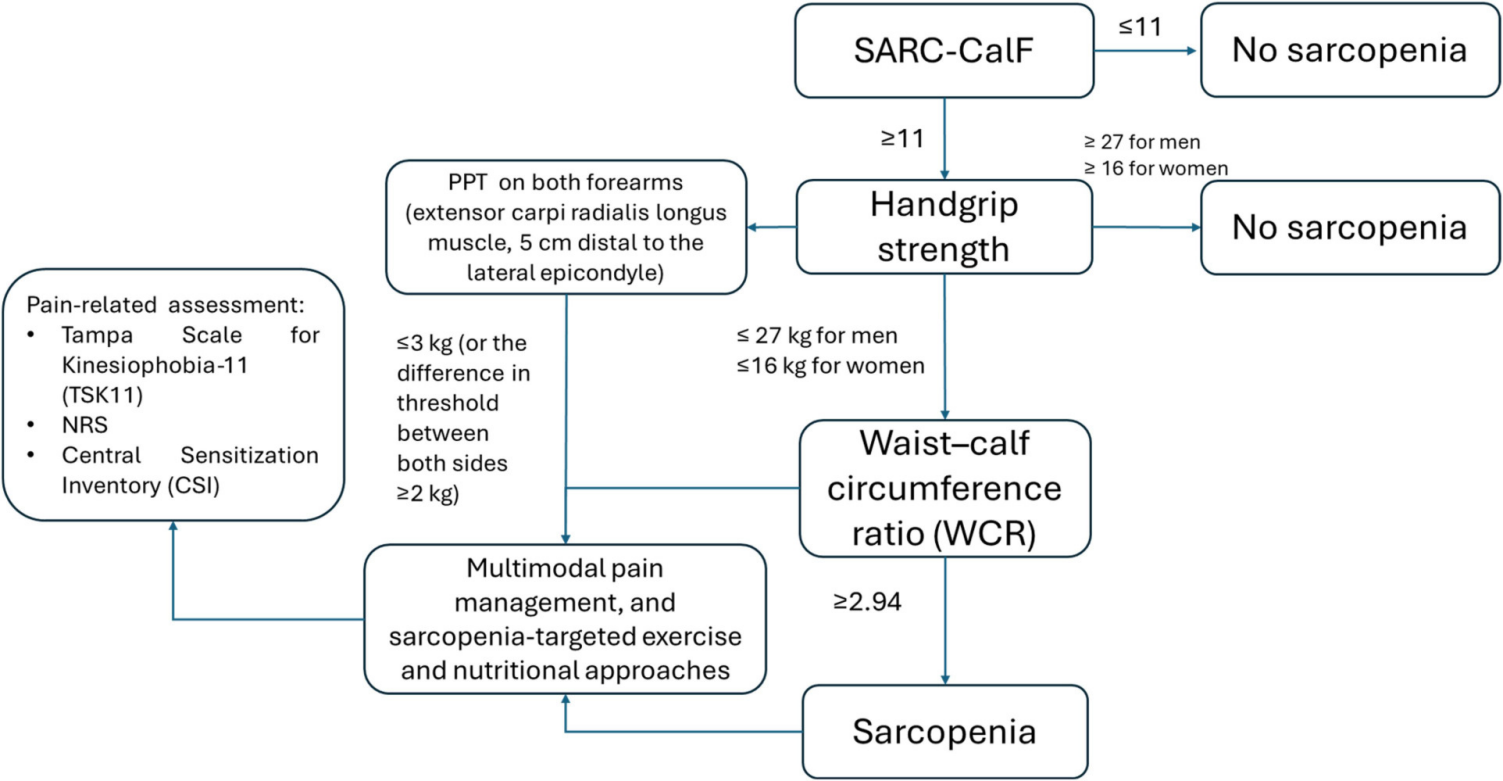
Management strategy for older people with sarcopenia and musculoskeletal pain.

Given the consistent epidemiological and mechanistic links between pain and sarcopenia, integration of pain assessment could be implemented without fundamentally altering existing diagnostic structures. At the screening level, validated tools such as the NRS and the Brief Pain Inventory (BPI) could be administered alongside SARC-F. Pain intensity and pain-related functional interference may help contextualize performance-based measures, reducing the risk of misclassification due to pain-limited effort. Pain phenotyping could support clinical interpretation of low muscle strength or poor physical performance. For example, documenting chronic musculoskeletal pain duration (>3 months), multisite pain, or features of central sensitization may identify individuals in whom muscle weakness and pain mutually reinforce functional decline. Rather than redefining sarcopenia, such integration would provide a complementary dimension that enhances clinical precision. A pragmatic approach could involve incorporating pain as a modifier or stratification variable. This would allow clinicians to distinguish between “pain-complicated sarcopenia” and sarcopenia without significant pain burden, potentially guiding tailored rehabilitation strategies. According to the available evidence on the bidirectional link between sarcopenia and pain, it may be hypothesized the application of a dedicated approach to better manage sarcopenia and musculoskeletal pain in older people (Figure 2).

## 5 Therapeutic overlap: sarcopenia-targeted strategies as pathways to pain relief

Clinical evidence addressing the sarcopenia–pain interface remains emerging and heterogeneous. Most available data derive from observational clinical studies or secondary analyses of rehabilitation cohorts, with a smaller number of interventional trials specifically targeting both muscle function and pain outcomes. Given the bidirectional relationship between sarcopenia and pain, therapeutic approaches must adopt an integrated, multimodal framework that simultaneously addresses muscle health and pain control. Clinical trials in older adults with chronic low back pain demonstrate that multicomponent exercise, together with nutritional strategies, represents the cornerstone of intervention, aiming to improve muscle mass, strength, and physical performance while also reducing pain and disability (51, 52). Nakagawa et al. (52) explored this complex interplay by enrolling elderly patients with chronic low back pain, stratified according to the presence or absence of sarcopenia. All participants underwent a standardized exercise therapy program—including stretching, strengthening, and postural training—and were evaluated for pain intensity, physical function, and quality of life. Importantly, sarcopenic patients achieved clinically significant improvements in functional recovery and pain relief, confirming that exercise remains a viable and effective intervention in this population. At the 3-month follow-up, both groups demonstrated a significant reduction in numeric rating scale (NRS) pain scores, alongside gains in trunk extensor strength, decreased disability as measured by the Roland-Morris Disability Questionnaire, and enhanced quality of life.

Experimental paradigms investigating exercise-induced hypoalgesia (EIH) provide further insight into therapeutic mechanisms. Observational and quasi-experimental studies indicate that sarcopenia may attenuate EIH responses, suggesting impaired descending inhibitory pathways. Yamaguchi et al. (53) examined whether EIH, the acute, endogenous pain-inhibitory effect following exercise driven by activation of descending inhibitory pathways, is impaired in older adults, and how such impairment relates to sarcopenia and descending pain modulation (conditioned pain modulation, CPM). Participants underwent standardized assessments of pain and skeletal muscle mass assessment before and after a bout of exercise. EIH was operationalized as the change in pain sensitivity (e.g., PPT increase) following the exercise stimulus, with CPM and skeletal muscle mass index (SMI) analyzed as potential contributors to the EIH response. Authors reported a blunted EIH response overall, indicating reduced exercise-evoked analgesia in older people, and sarcopenia was associated with further attenuation of EIH, suggesting that muscle wasting compromises the capacity of exercise to activate endogenous pain inhibition. The magnitude of EIH correlated positively with CPM and with SMI, while worse baseline pain sensitivity (lower PPT) related to smaller EIH gains. These findings suggest that assessing sarcopenia and CPM can help predict EIH responsiveness and guide individualized programs, for example by prioritizing progressive resistance exercise to bolster muscle reserve and graded aerobic training to engage descending inhibition (54). However, EIH was evaluated after a single exercise bout, therefore it cannot be excluded that regular exercise may partially restore EIH through neuroplastic changes in descending inhibition. Indeed, Vaegter & Jones (55) provided a broad overview of EIH, confirming that acute bouts of exercise reliably increase pain thresholds and reduce pain perception in healthy adults, while regular exercise programs (at least 2 months) lead to clinically meaningful reductions in pain intensity and disability across several chronic pain conditions. Taken together, evidence suggests that EIH is a robust but variable phenomenon, shaped by age, muscle status, and pain physiology, that in elderly sarcopenic patients remains present but attenuated.

Nutritional strategies, particularly adequate protein intake, vitamin D supplementation, and correction of micronutrient deficiencies, are essential to support muscle anabolism and reduce systemic inflammation (56) and are supported by experimental and small-scale clinical studies demonstrating improvements in muscle mass and modulation of inflammatory pathways. Micro- and macronutrients are modulators of pain and muscle health. For example, the role of vitamin D not merely for bone health but also for skeletal muscle function and regeneration, inflammatory modulation, and fall risk, has been widely investigated (57–60). Omega-3 fatty acids, Eicosapentaenoic acid (EPA) and docosahexaenoic acid (DHA), long-chain polyunsaturated fatty acids synthetized from-linolenic acid, are emerging players in this field, by modulating inflammaging, mechanistic target of rapamycin complex 1 (mTORC1) pathway, intracellular protein breakdown, mitochondrial biogenesis and function, amino acid transport, and neuromuscular junction activity (61). Amino acids, particularly leucine, are well known to stimulate muscle protein synthesis and sarcopenia-related clinical outcomes, but its role on pain modulation in sarcopenic patients has not been clinically investigated so far (62). However, Kato et al. (63) investigates the biological effects of leucine-enriched essential amino acids (LEAAs) on muscle recovery following eccentric contraction, a type of exercise known to induce significant muscle damage and pain, demonstrating that this approach modulates the inflammatory response while simultaneously supporting muscle repair, as confirmed by reduced markers of inflammation, such as interleukin-6, in rat muscle following eccentric exercise. This attenuation of inflammatory signaling is accompanied by enhanced muscle regeneration, suggesting that LEAAs strike a balance between allowing necessary immune activity and preventing secondary tissue damage and pain.

More recently, Vladutu et al. (64) supported the role of rehabilitation as the most effective way to manage sarcopenia and pain, also in obese people, by combining exercise, nutritional support, and psychosocial interventions into a single integrated framework. The authors showed that 12-week structured rehabilitation plan, including counselling about consequences of sarcopenia and obesity on functioning, emphasis on adequate protein intake (1.2–1.5 g/kg body weight/day, distributed evenly across meals), supplementation with vitamin D and calcium, multimodal exercise (i.e., muscle strengthening, balance and gait training, and aerobic training), physical modalities (i.e., pulsed electromagnetic field therapy, electrical muscle stimulation, low-level laser therapy, and vibration therapy), produces significant improvements of handgrip strength, pain, disability and quality of life.

Non-pharmacological strategies such as exercise and nutrition remain the cornerstone of sarcopenia management, yet the role of pharmacological interventions should not be underestimated. Pain is often a decisive barrier to participation in rehabilitation programs, and its effective control can make the difference between adherence and avoidance (65). In this regard, paracetamol (acetaminophen) emerges as a pragmatic option in older people with sarcopenia. Compared with non-steroidal anti-inflammatory drugs (NSAIDs), which are associated with gastrointestinal, renal, and cardiovascular risks, paracetamol offers a more favorable safety profile in older adults, making it a reasonable first-line choice (66). The strategic use of pharmacotherapy is also worth emphasizing scheduled administration prior to exercise sessions may prove more effective than on-demand use, as it anticipates pain and supports functional improvements (67). This proactive approach can help patients achieve the intensity and regularity of exercise necessary to induce muscle hypertrophy and improve physical performance function.

Beyond mechanical loading, skeletal muscle functions as an endocrine organ capable of secreting bioactive peptides known as myokines, such as myostatin (GDF-8) and irisin. These molecules regulate autocrine, paracrine, and endocrine signaling pathways that influence muscle metabolism, inflammation, and systemic homeostasis (68). Myostatin, a member of the TGF-β superfamily, inhibits muscle growth by suppressing satellite cell activation and protein synthesis via Smad signaling pathways. Elevated myostatin levels have been observed in sarcopenic populations (69). Preclinical models demonstrate that myostatin inhibition (e.g., via neutralizing antibodies, soluble receptors, or gene editing approaches) increases muscle mass and improves strength (70). Moreover, myostatin inhibition contributes to neuro-immune modulation that drives chronic musculoskeletal pain through nervous tissue macrophages, and its inhibition resolved pain and macrophage accumulation in the dorsal root ganglia (DRG) of animal model of knee osteoarthritis (71). However, translation into clinical trials has produced mixed results. Several myostatin inhibitors have shown increases in lean mass but limited functional improvements in older adults (72). Concerns regarding long-term safety and off-target effects remain under investigation. Thus, while biologically compelling, myostatin blockade has not yet demonstrated consistent clinical efficacy in sarcopenia mitigation as well as in sarcopenia-related pain.

Irisin, cleaved from FNDC5 during muscle contraction, promotes mitochondrial biogenesis via PGC-1*α* activation, thereby enhancing oxidative metabolism, reduces inflammatory signaling, and may improve NMJ stability (73). In an animal model of FNDC5/irisin knockout mice, irisin administration improves muscle mass and strength, and energy homeostasis (74). Moreover, it has been reported anti-inflammatory effects of irisin in a mouse model of inflammatory pain. In this study, irisin-treated mice exhibited a gradual reduction in mechanical allodynia and thermal hyperalgesia as well as an upregulation of the expression of M2 macrophage markers (IL-4, IL-10) and downregulation of M1 macrophage markers (IL-1β, IL-6, and TNF-α) at the site of pain and, at the DRG, and at the spinal cord (75). Nevertheless, clinical trials directly targeting irisin pathways remain limited (76), and its role as a therapeutic agent in sarcopenia and sarcopenia-related pain are still exploratory.

Clinical evidence supporting integrated interventions for sarcopenia and musculoskeletal pain remains heterogeneous, with variations in diagnostic criteria for sarcopenia, pain phenotyping, and intervention protocols. Randomized controlled trials specifically designed to target both conditions simultaneously are still limited. Overall, the current body of evidence supporting the sarcopenia–pain connection is strongest at the epidemiological level, biologically plausible at the mechanistic level, and promising but still evolving at the interventional level. Future research should prioritize longitudinal designs and targeted randomized trials to clarify causal pathways and optimize integrated therapeutic strategies.

## 6 Current limitations and future directions

Although substantial epidemiological and mechanistic evidence supports a bidirectional relationship between sarcopenia and chronic musculoskeletal pain, important clinical and translational gaps remain. Despite growing recognition of sarcopenia as a determinant of disability, available treatments remain only partially effective. Exercise is the cornerstone of therapy and consistently improves muscle strength. However, adherence is frequently limited in older adults, particularly in those with chronic pain, multimorbidity, or environmental constraints (77). Pharmacological approaches, including myostatin inhibitors and other anabolic agents, have shown increases in lean mass but inconsistent improvements in strength or physical performance (72). This mass–function discrepancy underscores a critical limitation of current therapeutic paradigms. Moreover, most interventional studies do not incorporate pain-related endpoints, despite the high prevalence of chronic musculoskeletal pain among sarcopenic individuals (78). This omission limits understanding of whether improvements in muscle parameters translate into meaningful symptom reduction. Finally, heterogeneity in diagnostic criteria and population selection complicates comparability across trials and may obscure treatment responsiveness.

Sarcopenia should not be viewed as a purely intrinsic biological consequence of aging. Rather, it emerges from dynamic interactions between molecular processes and environmental exposures (79). Physical inactivity, whether driven by sedentary lifestyle, chronic pain, fear of movement, or limited access to supportive environments, reduces mechanical loading, blunts mTOR signaling, decreases beneficial myokine release, and accelerates muscle atrophy (80). This inactivity further amplifies inflammatory signaling and mitochondrial dysfunction, creating a self-reinforcing cycle (81). Socioeconomic factors influence diet quality, healthcare access, and opportunities for physical activity, indirectly modulating biological vulnerability (82). Psychosocial stress, depression, and sleep disturbances, frequently observed in chronic pain populations, contribute to hypothalamic–pituitary–adrenal axis dysregulation, elevated cortisol levels, and impaired protein synthesis (83). Sarcopenia reflects the cumulative interaction between environmental determinants and intracellular pathways (79) involving inflammation, mitochondrial dysfunction, neuromuscular degeneration, and anabolic resistance. Recognizing these interactions has direct therapeutic implications. Exercise interventions exert effects not only through mechanical hypertrophy but also by modulating inflammatory pathways, enhancing mitochondrial function, stabilizing NMJ, and promoting beneficial myokine secretion (84). However, these biological benefits depend on environmental feasibility and behavioral adherence. Nutritional strategies must be individualized, accounting for socioeconomic context, appetite regulation, comorbidities, and anabolic responsiveness. Combined exercise–nutrition programs consistently outperform isolated interventions, reflecting synergy between mechanical and metabolic signaling (85). Pain management and psychological support may indirectly improve muscle outcomes by restoring activity levels, reducing stress-mediated catabolism, and improving sleep quality (86). Multidimensional rehabilitation models may therefore offer superior outcomes compared to isolated biological targeting.

To advance the field, several priorities should be addressed, including well-designed longitudinal cohort studies to clarify temporal and causal relationships between sarcopenia, chronic pain, and environmental exposures. These studies should integrate standardized diagnostic criteria (e.g., EWGSOP2, AWGS), detailed pain phenotyping, physical activity measures, nutritional assessment, and molecular biomarkers. Also, development and validation of pain-adjusted or context-sensitive screening strategies should be pursued. Randomized controlled trials should systematically incorporate pain outcomes and psychosocial variables as predefined endpoints. Interventions targeting muscle mass and strength should evaluate whether improvements translate into reductions in pain intensity, interference, and central sensitization. Mechanistically informed trials integrating molecular biomarkers (e.g., inflammatory cytokines, myokines, mitochondrial function markers) with environmental and behavioral interventions may help identify responder phenotypes and guide personalized therapy. Finally, future therapeutic paradigms should embrace multidimensional strategies combining resistance training, nutritional optimization, anti-inflammatory modulation, mitochondrial support, pain management, and environmental facilitation. Evaluating long-term sustainability and real-world implementation will be essential.

## 7 Conclusion

Sarcopenia and chronic musculoskeletal pain are not parallel phenomena but deeply interconnected conditions that reinforce one another through shared biological, mechanical, and psychosocial pathways. Pain accelerates muscle wasting by limiting mobility and fostering disuse, while sarcopenia increases vulnerability to painful syndromes through impaired biomechanics, inflammation, and neurophysiological sensitization. Although substantial epidemiological and mechanistic evidence supports a bidirectional relationship between sarcopenia and chronic musculoskeletal pain, several critical gaps remain. Longitudinal studies are needed to clarify temporal and causal relationships. Prospective cohort studies incorporating standardized sarcopenia criteria alongside detailed pain phenotyping would allow evaluation of whether pain independently predicts incident sarcopenia, or whether muscle decline increases pain vulnerability. Development and validation of pain-adjusted screening strategies represent a key priority. Existing sarcopenia screening tools may be influenced by pain-related disability, potentially leading to misclassification. Future research should explore modified screening algorithms incorporating brief validated pain measures, or stratification approaches distinguishing “pain-complicated sarcopenia” from sarcopenia without significant pain burden. Cross-cultural validation across diverse populations will be essential. Moreover, randomized controlled trials targeting sarcopenia should systematically incorporate pain outcomes as predefined endpoints. Exercise, nutritional, and multimodal rehabilitation interventions may exert dual effects on muscle function and nociceptive modulation. Including standardized pain intensity, interference, and central sensitization measures would clarify whether improvements in muscle strength translate into clinically meaningful pain reduction. Finally, translational studies integrating biological markers of inflammation, mitochondrial function, and neuromodulation could help identify shared therapeutic targets. A multidimensional framework that simultaneously addresses muscle health, biomechanical load, and pain processing may ultimately yield more effective and personalized interventions. Advancing this integrated research agenda may refine diagnostic pathways, improve functional outcomes, and reduce disability in aging populations affected by both sarcopenia and chronic pain.
